# C1q limits cystoid edema by maintaining basal **β**-catenin–dependent signaling and blood-retina barrier function

**DOI:** 10.1172/jci.insight.190227

**Published:** 2025-10-14

**Authors:** Lingling Zhang, Jacklyn Levey, Md. Abedin, Ha-Neul Jo, Emmanuel Odame, Miranda Howe, Kaia L. Douglas, Elise Thoreen, Scott W. McPherson, Heidi Roehrich, Somasekar Seshagiri, Stephane Angers, Zhe Chen, Harald J. Junge

**Affiliations:** 1 Department of Ophthalmology and Visual Neurosciences, University of Minnesota, Minneapolis, Minnesota, USA.; 2 Department of Biology, University of St. Thomas, Saint Paul, Minnesota, USA.; 3 Graduate Program in Molecular, Cellular, Developmental Biology and Genetics, University of Minnesota, Minneapolis, Minnesota, USA.; 4 AntlerA Therapeutics, Foster City, California, USA.; 5 Department of Biochemistry, University of Toronto, Toronto, Ontario, Canada.; 6 Terrence Donnelly Centre for Cellular and Biomolecular Research, Toronto, Ontario, Canada.; 7 Leslie Dan Faculty of Pharmacy, University of Toronto, Toronto, Ontario, Canada.; 8 Department of Neuroscience, University of Minnesota Medical School Twin Cities, Minneapolis, Minnesota, USA.

**Keywords:** Ophthalmology, Vascular biology, Complement, Endothelial cells, Lupus

## Abstract

Macular edema (ME) can cause profound vision impairment and occurs in several prevalent retinal diseases, including diabetic retinopathy, choroidal neovascularization, retinal vein occlusion, and uveitis. Retinal edema typically results from dysfunction of the blood-retina barrier (BRB), which is associated with increased retinal expression of complement components. It is unclear whether the classical complement pathway has detrimental or protective roles in the context of BRB dysfunction. Here, we characterized *Tspan12*-KO^DBM^ (disrupted BRB maintenance) mice, a mouse model of cystoid edema generated by genetically and pharmacologically manipulating β-catenin–dependent norrin/frizzled-4 (FZD4) signaling. We assessed BRB function, cystoid edema, electroretinogram, and microglia activation outcomes in an aging study with WT, *C1qa*-KO, *Tspan12*-KO^DBM^, and *Tspan12*-KO^DBM^*; C1qa*-KO compound mutant mice. Phenotypic analyses and cell-based experiments indicated that C1QA contributes to maintaining basal β-catenin–dependent signaling and that the absence of C1QA exacerbates BRB dysfunction, cystoid edema, and neuroinflammation in *Tspan12*-KO^DBM^*; C1qa*-KO compound mutant mice. Activation of β-catenin–dependent signaling by an anti-FZD4 and anti-LRP5 agonistic antibody modality achieved complete resolution of cystoid edema. This study shows that reducing or enhancing norrin/FZD4 signaling can increase or decrease cystoid edema, respectively, underscoring its potential as a therapeutic target in ME. Furthermore, this study provides insights into the contribution of C1QA to BRB maintenance.

## Abstract 2

This study introduces a mouse model of retinal cystoid edema, identifies roles of C1q, and shows that a FZD4/LRP5 agonist achieves resolution of cystoid edema.

## Introduction

Macular edema (ME) arises when the rate of fluid entry into the retina exceeds the rate of fluid export, resulting in a pathological accumulation of fluid. The macula is prone to the formation of edema, likely due to its anatomic specialization. Edema may appear as diffuse fluid accumulation associated with increased macular thickness, fluid accumulation in the subretinal space, or fluid in intraretinal cyst-like spaces (cystoid edema, CE). ME poses a significant risk to acute central vision and may lead to irreversible neural damage. This condition often develops as a complication of prevalent eye diseases, including diabetic retinopathy, age-related macular degeneration, retinal vein occlusion, and uveitis (1).

A common cause of ME is dysfunction of the inner blood-retina barrier (BRB), where vascular endothelial cells (ECs) play a pivotal role as a critical cellular component. The BRB maintains the retinal microenvironment by regulating transport at the interface of the circulatory system and the neural retina. ECs of an intact BRB are characterized by efficient tight junctions, low rates of transcytosis, close interactions with glial cells, a high degree of pericyte coverage, and the expression of transporters for nutrients, hormones, and metabolic waste (2). With an intact BRB, oncotic pressure (driving water flow into blood vessels toward the high concentration of plasma proteins) and hydrostatic pressure (driving water flow out of blood vessels) provide a degree of balance so that fluid extravasation does not overwhelm retinal export mechanisms into the vitreous or across the retinal pigment epithelium. With BRB breakdown and the resulting protein extravasation, oncotic pressure is reduced and fluid accumulation in the retina increases (3). In addition, protein extravasation (e.g., fibrinogen) causes inflammation (4).

The major pathway that induces and maintains BRB function is the norrin/frizzled-4 (FZD4) pathway. The secreted protein norrin (gene symbol *NDP*, for Norrie disease pseudoglioma) is released from Müller glial and horizontal cells and activates β-catenin–dependent signaling in ECs by binding to a receptor complex containing the membrane proteins FZD4, low-density lipoprotein receptor–related 5 (LRP5), and tetraspanin 12 (TSPAN12). This signaling pathway plays a crucial role in enabling retinal angiogenesis, BRB induction, and BRB maintenance (5–7). The loss of norrin signaling in mice can cause CE, even in the absence of a macula (8). However, norrin-gene-disrupted mice are characterized by compounding pathologies, including vascular malformations and hypoxia (9), complicating the analysis of pathological roles of BRB dysfunction in CE. TSPAN12 is a coreceptor for norrin (10, 11) and is required for norrin/FZD4 signaling (12). *Tspan12* EC-specific-knockout (ECKO) mice have been used to separate angiogenesis and BRB defects by inducing tamoxifen-induced recombination after angiogenesis is complete. While this model circumvents the occurrence of vascular malformations and hypoxia, CE in *Tspan12*-ECKO mice is variable (7). Improved mouse models that display extensive CE despite the lack of a macula are needed. This is important for better understanding of CE and to test therapeutic approaches. For example, the activation of the norrin/FZD4 pathway by agonistic antibodies that bind FZD4 and LRP5 or FZD4 and LRP6 (13–15) alleviate BRB defects and suppress neovascularization in oxygen-induced retinopathy models (16–18). FZD4/LRP5 and FZD4/LRP6 agonists emerge as a class of agonists with potential uses in ME, but efficacy in preclinical mouse models of retinal edema remains to be demonstrated.

We previously reported that complement components are elevated in mice with impaired BRB maintenance (7). This finding sparked questions about the role of complement in the context of BRB dysfunction. The classical complement pathway is part of the innate immune system and is implicated in retinal disease (19). The C1 complex starts the classical complement cascade by activation of C1r and C1s serine proteases. C1q (which is composed of 6 heterotrimers, each containing one C1QA, C1QB, and C1QC polypeptide) serves as an essential scaffold in the C1 complex (20). Upon encountering antigen-antibody complexes or other activators, e.g., phosphatidyl serine on apoptotic cells (21) or pentraxins (22), the C1 complex initiates a cascade that can lead to cell destruction via the membrane attack complex, or cell opsonization and phagocytosis (23). C1q binds to multiple types of complement receptors to promote phagocytosis (24).

The classical complement system is important for maintaining tissue homeostasis, e.g., in synapse elimination (25) and wound healing (26). Pathophysiological actions include roles in neurodegenerative disease (27, 28), glaucoma (29), and in autoimmune diseases (30). Among the latter, C1q deficiency is a major risk factor for systemic lupus erythematosus (SLE) (31), as insufficient clearance of apoptotic cells and nuclear antigens promotes autoantibody generation in SLE (32). Immunoglobulin extravasation in the retina due to BRB dysfunction could further promote autoimmunity in C1q deficiency. Whether *C1qa*-KO mice develop retinal manifestations of SLE (including hemorrhages, and cotton-wool spots) when C1q deficiency is compounded with strong BRB defects and immunoglobulin extravasation, is not known.

The classical complement pathway is implicated in retinal diseases characterized by BRB dysfunction (19). Furthermore, increased retinal complement component expression was observed in mice with BRB dysfunction (7). This raises the question whether BRB dysfunction drives retinal disease progression via the classical complement pathway. Abundant extravasated immunoglobulin could form antigen-antibody complexes on the cell surface of retinal cells that activate the classical complement pathway, which may enhance phagocytosis and cause retinal damage. However, protective roles for C1q are also plausible. C1-dependent cleavage of LRP6 and activation of β-catenin–dependent signaling was reported in skeletal muscle regeneration and arterial remodeling (33, 34). Therefore, C1q could be required for BRB maintenance by promoting basal β-catenin–dependent signaling in ECs. Thus, whether the classical complement pathway in the context of BRB dysfunction has detrimental or protective roles remains poorly understood.

Here, we establish a mouse model of BRB maintenance defects and CE based on genetically and pharmacologically manipulating norrin/FZD4 signaling at specific stages of retinal vascular development. We use this model to test the role of the classical complement system in the context of BRB breakdown. Interestingly, we find that the loss of C1QA exacerbates BRB dysfunction and associated pathologies. Results from cell-based studies indicate a role of C1q in maintaining basal levels of β-catenin–dependent signaling in ECs, revealing a role of C1q in BRB maintenance. Furthermore, our analysis shows that increasing or decreasing norrin/FZD4 signaling modulates CE. We find that FZD4/LRP5 agonists alleviate BRB dysfunction and completely resolve CE, highlighting that norrin/FZD4 signaling is a highly suitable target for pharmacological intervention in ME.

## Results

### Tspan12-KO^DBM^ mice display extensive CE.

**Figure 1:**
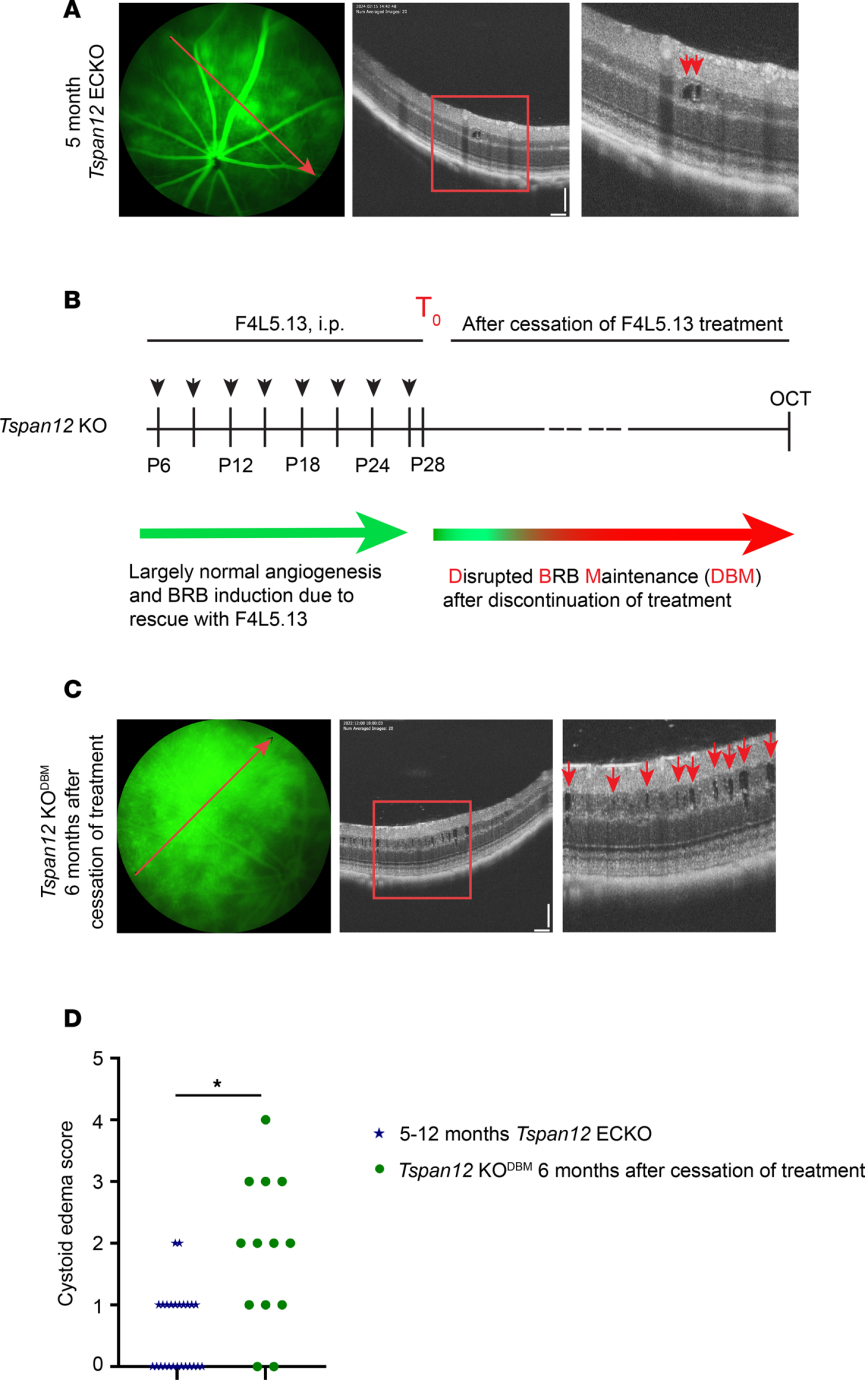
CE is more severe in *Tspan12*-KO^DBM^ mice compared with *Tspan12*-ECKO mice. (**A**) Representative FA fundus images and OCT scan images show *Tspan12*-ECKO mice with spotty retinal vascular leakage and moderate CE lesions. The red lines show the OCT line scan relative to the FA image. Boxed areas are shown enlarged in the panels on the right. Red arrows point to CE lesions. Scale bars: 100 μm. (**B**) Schematic representation of F4L5.13 administration to *Tspan12*-KO mice until P28 (T_0_) and subsequent cessation of treatment to generate *Tspan12*-KO^DBM^ (disrupted BRB maintenance) mice. (**C**) Representative FA fundus images and OCT scan images show *Tspan12*-KO^DBM^ mice with extensive CE. Scale bars: 100 μm. (**D**) CE scores in *Tspan12*-ECKO mice and *Tspan12*-KO^DBM^ mice. *n* = 13 *Tspan12*-KO^DBM^ and *n* = 21 *Tspan12*-ECKO retinas. A Mann-Whitney nonparametric test was used to test for differences in data on an ordinal scale. *P < 0.05.

*Tspan12*-ECKO mice develop and maintain a normal deep vascular plexus if tamoxifen-dependent recombination is induced after the phase of retinal developmental angiogenesis is over. While the 3-layered retinal vasculature is maintained, *Tspan12*-ECKO mice exhibit BRB defects and moderate CE due to impaired norrin/FZD4 signaling (7). We found that this model was useful to correlate defined leakage areas directly with sites of CE formation using fluorescein angiography–guided (FA-guided) optical coherence tomography (OCT) (Figure 1A). However, Cre-mediated recombination in *Tspan12*-ECKO mice is not complete (18), resulting in only mild or moderate CE that is not fully penetrant (Figure 1, A and D). To generate a model with increased and more penetrant CE, we sought to use a model with complete *Tspan12* gene inactivation while bypassing vascular malformations and hypoxia, which are characteristic phenotypes of *Tspan12*-KO mice. We used a strategy of pharmacologically and genetically manipulating norrin/FZD4 signaling at specific developmental stages to achieve that. Angiogenesis and BRB phenotypes of *Tspan12*-KO mice were rescued by administration of F4L5.13, an anti-FZD4/anti-LRP5 agonistic antibody and norrin mimetic, every 3 days from P6 to P28, as described previously (18). The administration of F4L5.13 during postnatal development supports virtually normal retinal angiogenesis and formation of the BRB in *Tspan12*-KO mice (18). However, after cessation of treatment, norrin/FZD4 signaling is no longer activated and therefore the maintenance of the BRB is disrupted (schematic in Figure 1B). Hereafter, we refer to this model as *Tspan12* KO^DBM^, for disrupted BRB maintenance. Six months after cessation of treatment, *Tspan12*-KO^DBM^ mice displayed severe BRB dysfunction and widespread CE (Figure 1C). We assigned each retina a CE score ranging from 0 to 5 using a grading scale described in Supplemental Figure 1; supplemental material available online with this article; https://doi.org/10.1172/jci.insight.190227DS1 This analysis revealed that the vast majority of *Tspan12*-KO^DBM^ mice displayed CE, whereas CE in *Tspan12*-ECKO mice was sporadic and milder (Figure 1D). CE in both models was predominantly detected in the inner nuclear layer, correlating with the high density of leaky intraretinal capillaries that flank the inner nuclear layer on both sides. Thus, by genetic and pharmacological manipulation of norrin/FZD4 signaling, we created a mouse model with extensive CE.

### Tspan12-KO^DBM^; C1qa-KO compound mutant mice display increased retinal vascular leakage and CE compared with Tspan12-KO^DBM^ mice.

**Figure 2:**
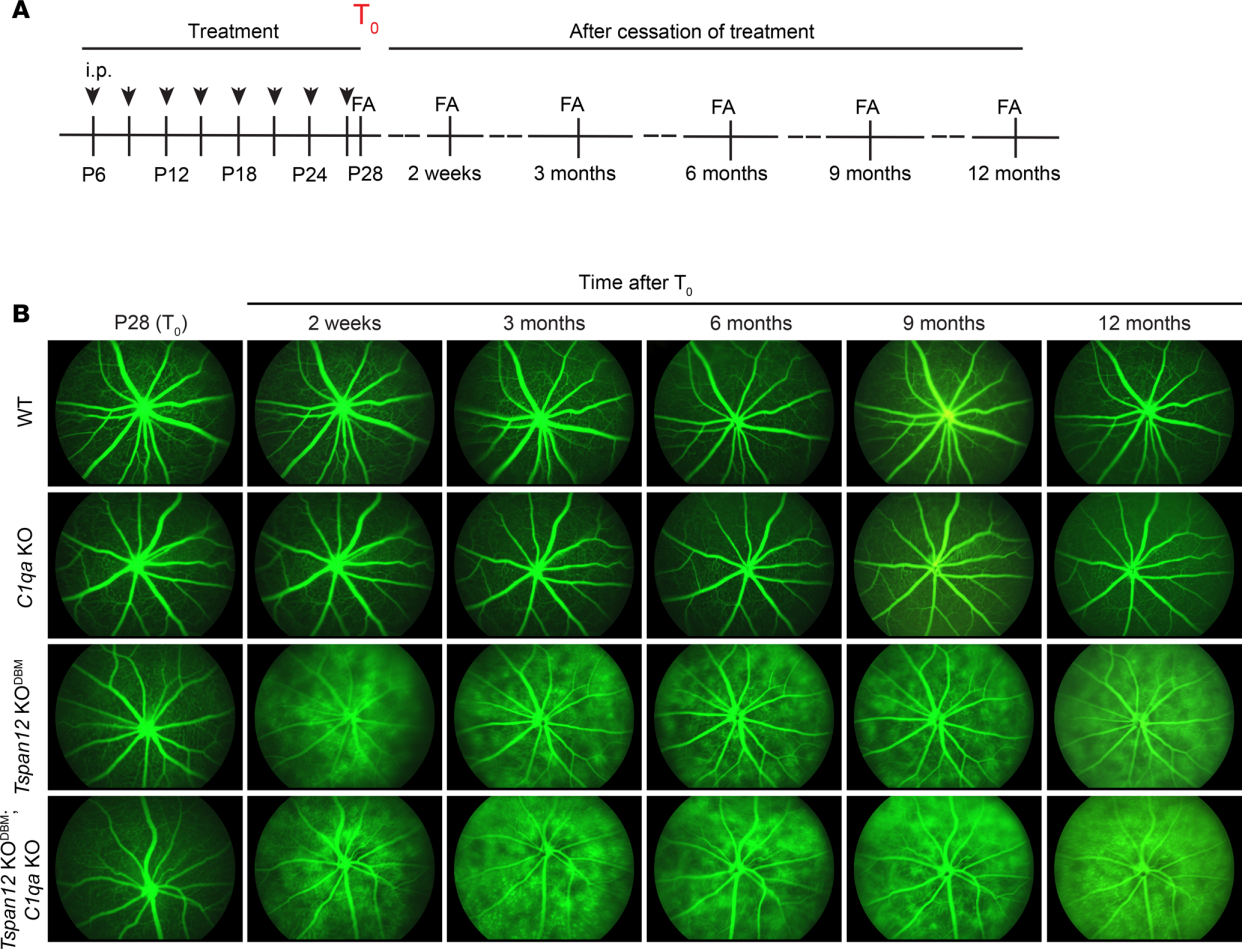
Longitudinal FA imaging in 4 groups of mice. (**A**) Schematic overview of the longitudinal study design. (**B**) The images are representative of 5–8 mice per group with similar results.

Next, we used this model of CE to better define the role of the classical complement system in the context of BRB dysfunction. To inactivate the classical complement pathway, we used a *C1qa*-null allele (35), as C1QA is a structural component of the C1 complex necessary for the activation of the classical complement cascade (20). Four groups of mice were followed in a year-long longitudinal FA study to assess BRB function: WT, *C1qa*-KO, *Tspan12*-KO^DBM^, and *Tspan12*-KO^DBM^; *C1qa*-KO compound mutant mice. *Tspan12*-KO^DBM^ mice were generated by repeated administration of F4L5.13 until P28, which was defined as time point T_0_ (Figure 2A). FA at P28 confirmed our previous report that F4L5.13 rescues the BRB phenotypes of *Tspan12*-KO mice (18). At T_0_ + 2 weeks, the BRB was already substantially compromised due to cessation of treatment. There was no notable leakage in the retinas of *C1qa*-single-KO mice, which appeared comparable to WT control. *Tspan12*-KO^DBM^; *C1qa*-KO compound mutant mice exhibited increased leakage compared with *Tspan12*-KO^DBM^ mice; however, nonquantitative FA was not sufficient to ascertain this difference (Figure 2B). Therefore, we quantified sulfo-NHS-LC-biotin leakage (a terminal procedure) in all 4 groups at the T_0_ + 12-month endpoint of the study.

**Figure 3:**
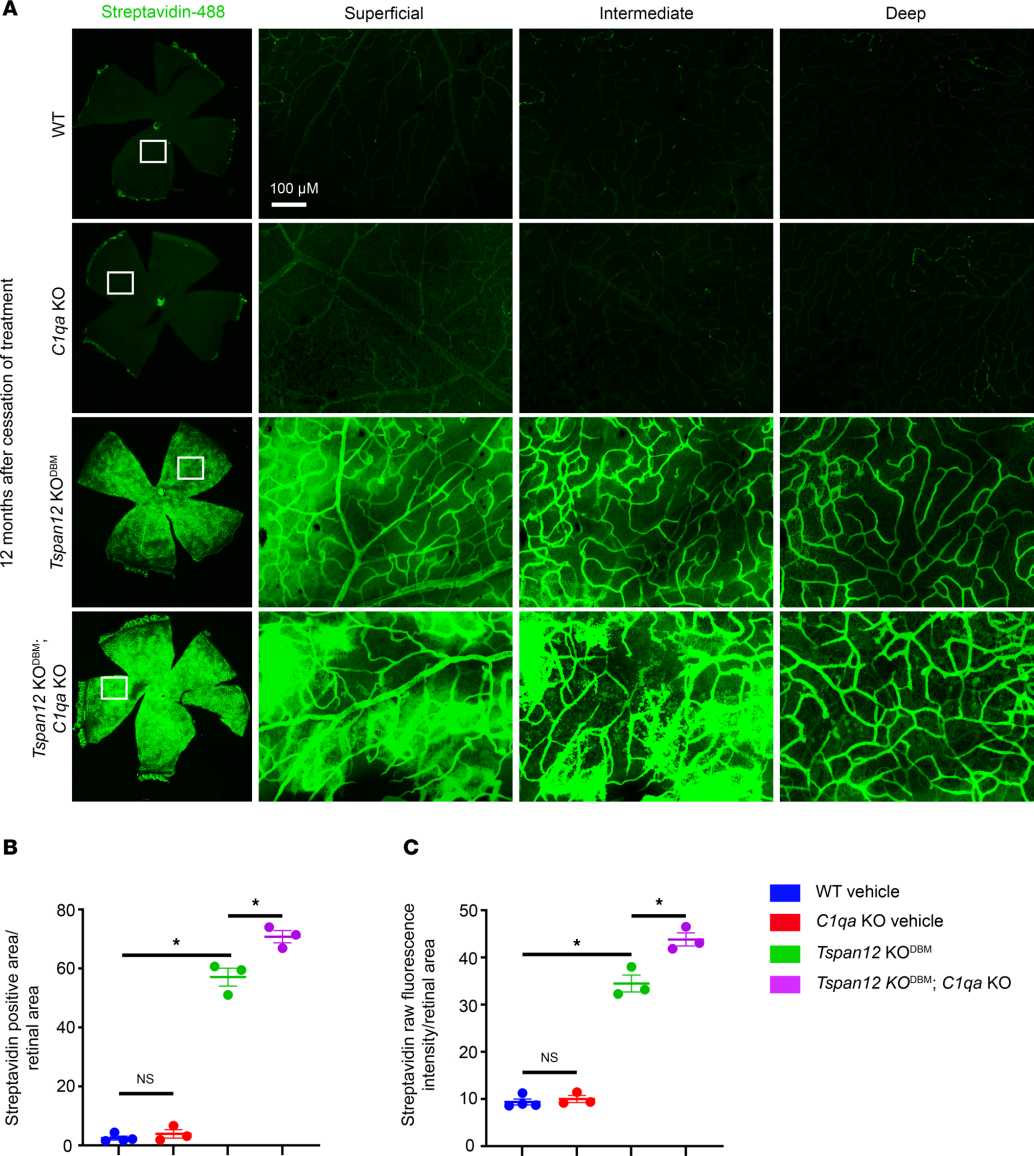
Increased sulfo-NHS-LC-biotin tracer leakage in *Tspan12*-KO^DBM^; *C1qa*-KO compound mutant retinas compared with *Tspan12*-KO^DBM^ retinas. (**A**) Left panels: Stitched ×4-magnified images of flat mount retinas. Right panels: ×20-magnified projections of the areas demarcated by white boxes. 3D image stacks were acquired and projections for each of 3 vascular layers were generated. Image acquisition settings were optimized for the strong signal in *Tspan12*-KO^DBM^ mice. Scale bar: 100 μm. (**B**) Streptavidin-positive area above the threshold was quantified. Four areas per retina were imaged and the data from each retina was averaged. *n* = 3–4 retinas per group. Average ± SEM is shown. (**C**) Quantification as described in **B** based on raw fluorescence intensity per retinal area. *P < 0.05 by 1-way ANOVA with Tukey’s post hoc test.

**Figure 4:**
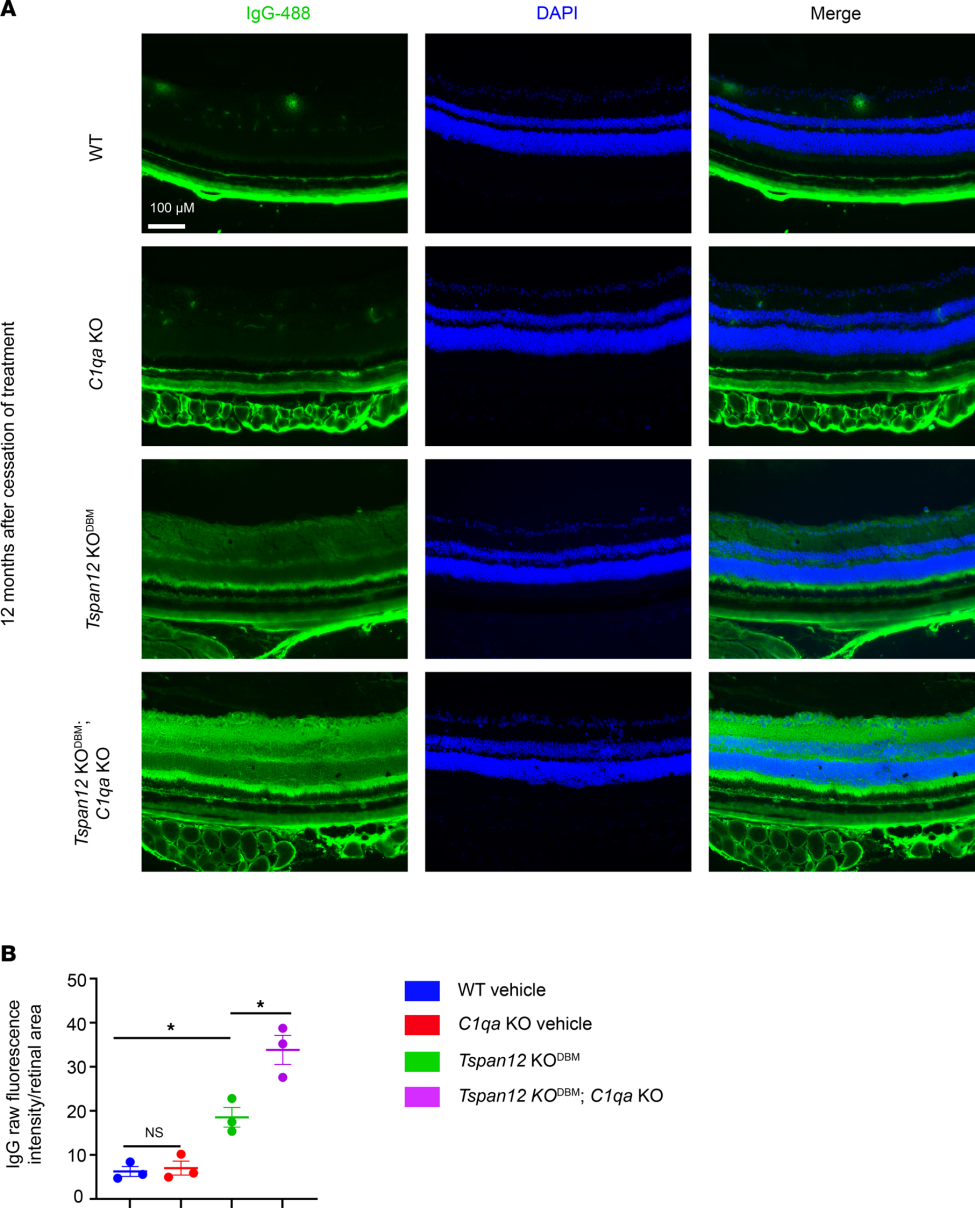
Increased IgG extravasation in *Tspan12*-KO^DBM^; *C1qa*-KO compound mutant retinas compared with *Tspan12*-KO^DBM^ retinas. (**A**) Retinal sections stained with anti-IgG and DAPI. Scale bar: 100 μm. (**B**) Quantification of IgG intensity in the retina from *n* = 3 retinas from 3 mice. Average ± SEM is shown. *P < 0.05 by 1-way ANOVA with Tukey’s post hoc test.

Sulfo-NHS-LC-biotin is a reactive tracer that biotinylates plasma proteins and luminal retinal EC proteins while it circulates through the vasculature. When the BRB is impaired, the tracer leaks out of retinal blood vessels and biotinylates proteins in the vascular basement membrane, perivascular space, and retinal parenchyma. Covalently attached biotin is subsequently detected using fluorescent streptavidin probes. Unlike diffusible FITC, biotin signal is not attenuated during wash steps and is therefore less variable. We used this approach to quantify BRB leakiness in 12-month-old mice from retinal whole mounts, which were imaged using parameters optimized for the strong signal in *Tspan12*-KO^DBM^ mice and *Tspan12*-KO^DBM^; *C1qa*-KO compound mutant mice. This experiment revealed a moderate but statistically significant increase in tracer extravasation in *Tspan12*-KO^DBM^; *C1qa*-KO compound mutant mice compared with *Tspan12*-KO^DBM^ mice (Figure 3, A–C). Leakiness was further evaluated by staining retinal sections with anti-IgG to monitor IgG extravasation and accumulation in the retina. This analysis confirmed more severe BRB dysfunction in *Tspan12*-KO^DBM^; *C1qa*-KO compound mutant mice compared with *Tspan12*-KO^DBM^ mice (Figure 4, A and B).

**Figure 5:**
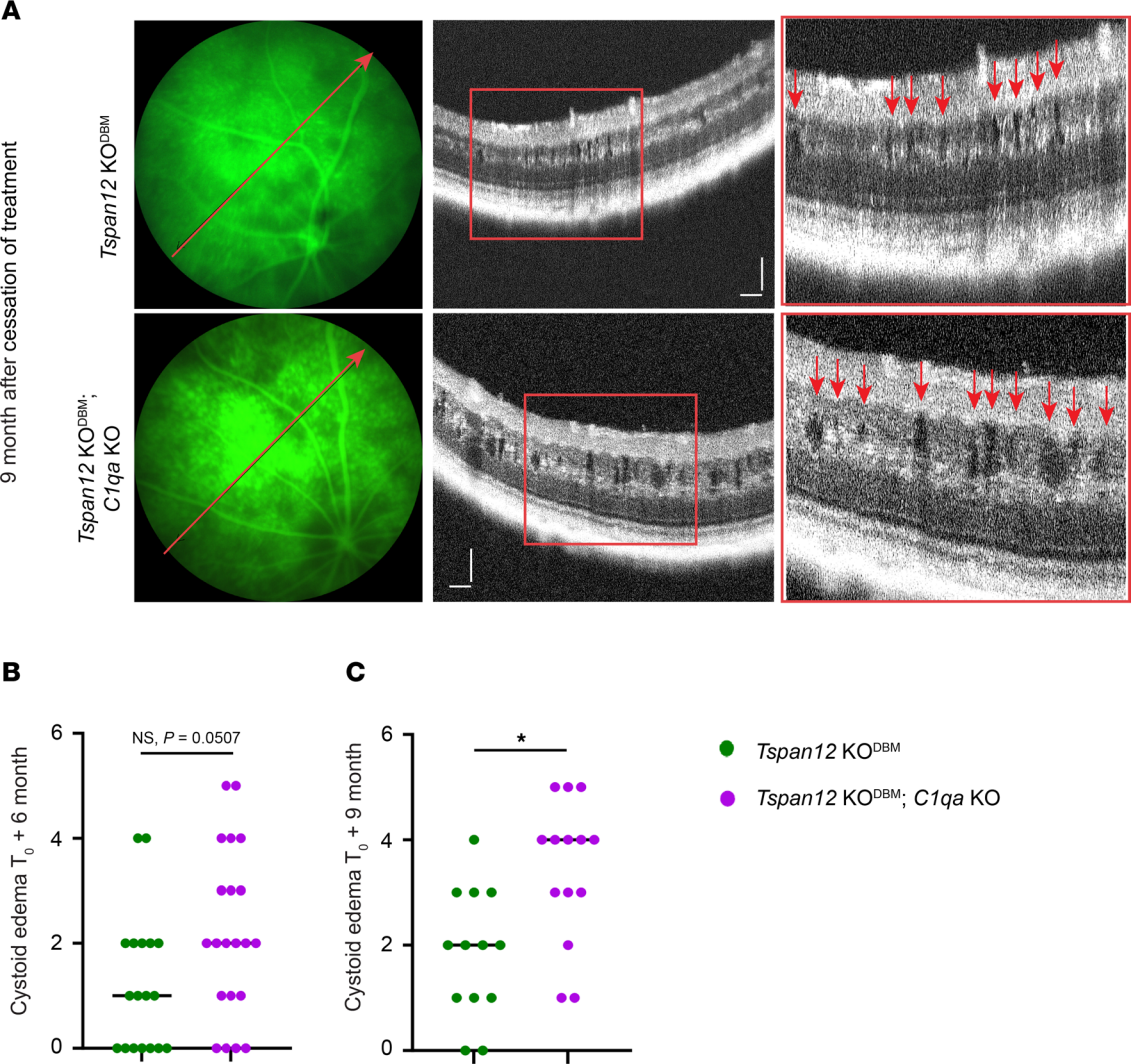
Increased CE in *Tspan12*-KO^DBM^; *C1qa*-KO compound mutant retinas compared with *Tspan12*-KO^DBM^ retinas. (**A**) Representative FA fundus images and OCT scan images. The red lines show the OCT line scan relative to the FA image. Boxed areas are shown enlarged in the panels on the right. Red arrows point to CE lesions. Scale bars: 100 μm. (**B**) At T_0_ + 6 months, CE scores in the *Tspan12*-KO; *C1qa-*KO mice tended to be higher than in the *Tspan12*-KO mice but did not reach significance. *n* = 18–21 retinas. (**C**) At T_0_ + 9 months, a significant difference in CE scores was observed. *n* = 13–14 retinas. *P < 0.05 by Mann-Whitney nonparametric test, as the CE scores are on an ordinal scale.

Six to 9 months after cessation of treatment with F4L5.13, substantial BRB leakage and CE were observed in the retinas of *Tspan12*-KO^DBM^ mice and *Tspan12*-KO^DBM^; *C1qa*-KO compound mutant mice. In most compound mutant mice, CE appeared more severe than in single mutant *Tspan12*-KO^DBM^ mice (Figure 5A). Grading the CE severity revealed that at T_0_ + 6 months, CE scores in compound mutant mice tended to be higher than in single mutant mice but did not quite reach significance (*P* = 0.0507, Figure 5B). At T_0_ + 9 months, CE scores of compound mutant mice were significantly higher than those of single mutant mice (Figure 5C). Thus, the severity of CE scores correlated with the increased severity of sulfo-NHS-LC-biotin leakage in *Tspan12*-KO^DBM^; *C1qa*-KO compound mutant mice compared with *Tspan12*-KO^DBM^ single mutant mice. C1q protein levels in serum and CNS rise substantially with age (33, 36), which correlates with our finding of increasing phenotypic differences between *Tspan12*-KO^DBM^ and *Tspan12*-KO^DBM^; *C1qa*-KO retinas during aging.

### ERG b-wave defects in Tspan12-KO^DBM^ and Tspan12-KO^DBM^; C1qa-KO mice.

**Figure 6:**
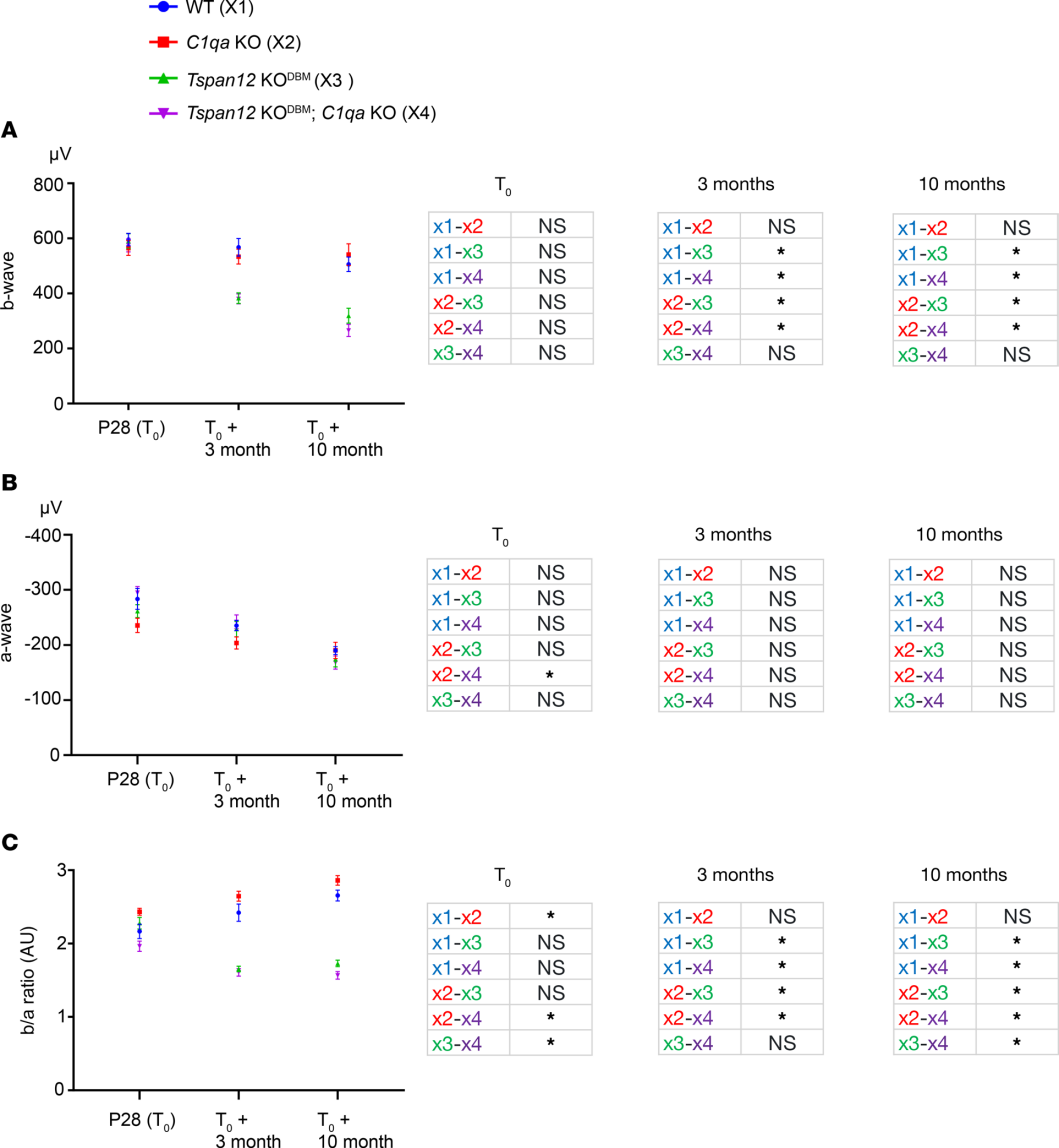
ERG b-wave reduction in mice with BRB maintenance defects. (**A**) P28: *n* = 16–24 retinas. T_0_ +3 months: *n* = 16–24 retinas. T_0_ +10 months: *n* = 14–16 retinas. Error bars represent SEM. (**B**) ERG a-wave of the same mice as detailed above. Average ± SEM shown. (**C**) The b/a amplitude ratio of the same mice as detailed above. Average ± SEM shown. *P < 0.05 by 1-way ANOVA with Tukey’s post hoc test. At the T_0_ + 3-month time point, a nonparametric Kruskal-Wallis test was used due to unequal variances.

We used dark-adapted electroretinography to examine retinal functional differences at 3 time points: T_0_, T_0_ + 3 months, and T_0_ + 10 months. As expected, electroretinogram (ERG) a-wave and b-wave amplitudes in all groups declined with age (37). The b-wave reflects the net effect of ion currents across the membranes of inner retinal cell populations, predominantly bipolar cells, in response to rod activity. The a-wave reflects the net effect of ion currents across the membranes of the rod cell population. At T_0_, the b-wave amplitude of *Tspan12*-KO^DBM^ mice compared to WT mice was not significantly changed, because repeated administration of F4L5.13 rescues postnatal retinal angiogenesis and restores the scotopic ERG b-wave in *Tspan12*-KO mice (18). However, a striking decrease in the b-wave amplitude of *Tspan12*-KO^DBM^ and *Tspan12*-*KO^DBM^*; *C1qa*-KO compound mutant mice was observed at the T_0_ + 3-month and T_0_ + 10-month time points compared with both control genotypes (Figure 6A). This strong reduction in the ERG b-wave correlated with the strong BRB defects and CE in *Tspan12* single and compound mutant mice. In contrast, the dark-adapted a-wave was not significantly changed (Figure 6B). Accordingly, the b/a amplitude ratio (expressed as absolute value) was significantly lower in *Tspan12*-KO^DBM^ and *Tspan12*-KO^DBM^; *C1qa*-KO compound mutant mice at T_0_ + 3 months and T_0_ + 10 months compared with both control genotypes (Figure 6C). While the b/a ratio tended to be smaller in *Tspan12*-KO^DBM^; *C1qa*-KO compound mutant mice compared with *Tspan12*-KO^DBM^ single mutant mice at early time points, this difference became significant at the T_0_ + 10-month time point, consistent with the increased sulfo-NHS-LC-biotin leakage and CE in the compound mutant group. In addition, we observed a significant increase in the b/a ratio in *C1qa*-KO mice compared with WT mice at T_0_ and a nonsignificant trend toward higher b/a ratios at the later time points, which may reflect developmental differences at the photoreceptor triad synapse in *C1qa*-KO mice (38). We note that increased b/a ratios due to loss of C1QA may lead to an underestimation of the reduction of b/a ratios in *Tspan12*-KO^DBM^; *C1qa*-KO compound mutant mice compared with *Tspan12*-KO^DBM^ single KO mice.

### Microglia and macroglia phenotypes in Tspan12-KO^DBM^ and Tspan12-KO^DBM^; C1qa-KO mice.

**Figure 7:**
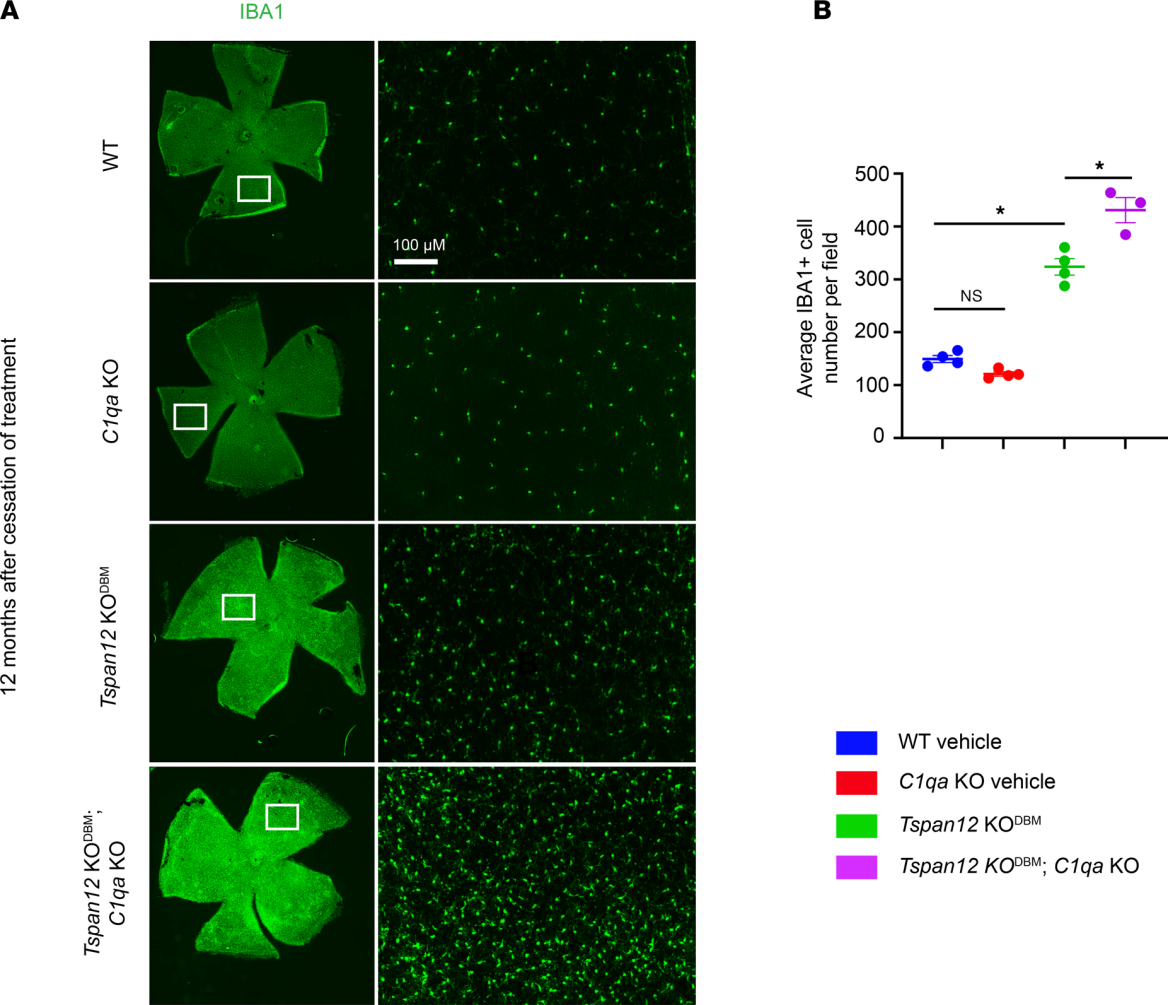
Microglia and macroglia phenotypes in mice with BRB maintenance defects. (**A**) Shown are ×4-magnified stitched images of whole-mount retinas stained for the microglia/monocyte-derived macrophage marker IBA1. Boxed areas are imaged at ×20 magnification and shown in the right panel. Scale bar: 100 μm. (**B**) Quantification of IBA1^+^ cells per ×20-magnified field of view. *n* = 3–4 retinas per group. Average ± SEM shown. *P < 0.05 by 1-way ANOVA with Tukey’s post hoc test.

Endothelial blood-CNS barrier breakdown triggers neuroinflammation, for example due to the extravasation of fibrinogen (4). Conversely, neuroinflammation is a cause for barrier dysfunction. This reciprocal relationship can lead to amplification of both barrier dysfunction and neuroinflammation (39). Expansion of microglia or infiltration of monocyte-derived macrophages can be a feature of neuroinflammation. We stained for IBA1, a marker of resident retinal microglia and monocyte-derived macrophages, after tissue was harvested at the T_0_ + 12-month endpoint of the study. We found a strong IBA1^+^ microglia/macrophage cell expansion in *Tspan12*-KO^DBM^ and *Tspan12*-KO^DBM^; *C1qa*-KO compound mutant mice (Figure 7A). Expansion of IBA1^+^ cells was more severe in the compound mutant mice (Figure 7B). These data were corroborated by quantifying the intensity of myeloid cell marker CD11b from retinal whole mounts (Supplemental Figure 2, A and B). Consistent with the quantification of myeloid cell markers, we found a significant increase in *Trem2* mRNA, a marker for microglia/monocyte-derived macrophages. *Trem2* was increased in *Tspan12*-KO^DBM^ and *Tspan12*-KO^DBM^; *C1qa*-KO compound mutant mice; this increase tended to be more pronounced in *Tspan12*-KO^DBM^; *C1qa*-KO compound mutant mice compared with *Tspan12*-KO^DBM^ single mutant mice, although the difference did not reach significance (*P* = 0.11) (Supplemental Figure 3A). TREM2 is a context-dependent modulator of myeloid cell behavior and the increase in *Trem2* in *Tspan12*-KO^DBM^ retinas and *Tspan12*-KO^DBM^; *C1qa*-KO compound mutant retinas may correlate with the increased need for phagocytosis in the context of BRB dysfunction.

How glia respond to endothelial blood-CNS barrier dysfunction is incompletely understood. We found that the mRNAs of 2 retinal glia markers, *C4b* (Supplemental Figure 3B) and *Aqp4* (Supplemental Figure 3C) were upregulated in *Tspan12*-KO^DBM^ and *Tspan12*-KO^DBM^; *C1qa*-KO compound mutant mice. C4b may help in the clearance of extravasated protein or cell debris via opsonization (40) and is expressed in retinal astrocytes and activated microglia (9). AQP4, which is expressed in Müller cells and astrocytes, is involved in water export from the retina (3) and may partially compensate for the increased fluid entry through a leaky BRB. GFAP was strongly expressed in astrocytes; in addition, Müller cells expressed GFAP in *Tspan12*-KO^DBM^ and *Tspan12*-KO^DBM^; *C1qa*-KO compound mutant retinas, indicating reactive gliosis (Supplemental Figure 4).

BRB dysfunction, myeloid cell changes, and reactive gliosis were not sufficient to cause a detectable increase in apoptosis in 12-month-old *Tspan12*-KO^DBM^ and *Tspan12*-KO^DBM^; *C1qa*-KO compound mutant retinas, while a *tert*-butyl hydroperoxide–treated retina processed in parallel served as a technical positive control to ensure efficient terminal deoxynucleotidyl transferase dUTP nick-end labeling (TUNEL) (Supplemental Figure 5). These data imply that the reduced ERG b-wave is not associated with substantial loss of retinal cells.

Although C1q deficiency can lead to SLE with retinal manifestations in human patients, e.g., hemorrhages and microinfarctions in the nerve fiber layer (cotton-wool spots) (41), such phenotypes have not been reported in the retinas of *C1qa*-KO mice. Because immunoglobulin extravasation in *Tspan12*-KO^DBM^; *C1qa*-KO compound mutant mice may promote autoimmunity, we examined the fundus of mice of all 4 genotypes. However, immunoglobulin extravasation and C1q deficiency was not sufficient to cause hemorrhages or cotton-wool spots (Supplemental Figure 6) on a C57BL/6J background (see Discussion).

### Loss of C1QA dampens ligand-independent basal β-catenin–dependent signaling.

**Figure 8:**
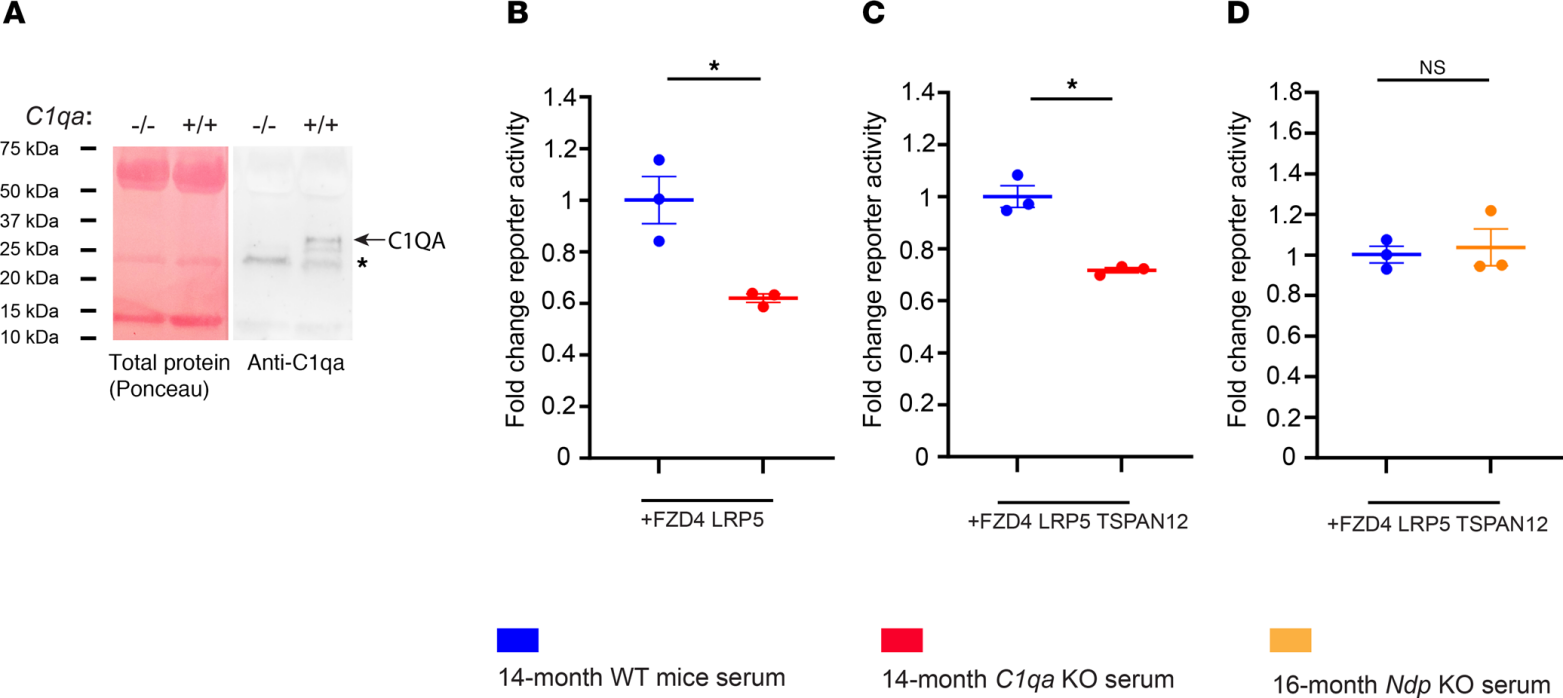
Loss of C1QA dampens ligand-independent basal β-catenin–dependent signaling through FZD4. (**A**) Western blot detects C1QA in serum of WT mice. Asterisk indicates a nonspecific band. Total protein (using Ponceau S stain) of the same blot shown in the left panel. Data are representative of 3 samples from 3 mice per genotype. (**B**–**D**) TOPFlash Dual-Glo assay (firefly activity divided by Renilla activity) in 293T cells transfected with the indicated constructs. Cells were cultured in serum from mice of different genotypes as indicated in the figure. *n* = 3 biological replicates, average ± SEM shown. *P < 0.05 by Welch’s heteroscedastic *t* test.

In skeletal muscle regeneration and arterial remodeling, complement C1q promotes basal β-catenin–dependent signaling in a Wnt-independent manner by binding to frizzled receptors and inducing C1s-dependent cleavage of the ectodomain of the Wnt coreceptor LRP6 (33, 34). We wondered whether loss of C1QA in *Tspan12*-KO^DBM^; *C1qa*-KO compound mutant mice reduces basal β-catenin signaling in ECs in a context where ligand-induced norrin/FZD4 signaling is already strongly impaired. A reduction in basal signaling could explain the exacerbated BRB leakage and increased CE in *Tspan12*-KO^DBM^; *C1qa*-KO compound mutant mice compared with *Tspan12*-KO^DBM^ mice. To test this hypothesis, we performed TOPFlash luciferase reporter assays using 293T cells transfected with FZD4 and LRP5. Cells were cultured in medium with serum from aged WT mice, a major source of C1q (33), or serum from age-matched *C1qa*-KO mice. Western blot analysis of serum from WT and *C1qa*-KO mice showed that C1QA expression was disrupted as expected (Figure 8A). Basal signaling activity (without norrin stimulation) was significantly decreased in cells cultured in C1QA-deficient medium compared with medium containing serum from WT mice (Figure 8B), consistent with a previous report that used WT versus *C1qa*-KO serum to stimulate basal signaling in other biological contexts (33). Basal reporter activity was also reduced in cells coexpressing the TSPAN12 coreceptor for norrin (Figure 8C). To rule out the possibility that norrin contributed to activating the TOPFlash reporter, we compared TOPFlash activity of cells cultured in medium with WT serum versus serum from *Ndp*-KO mice and found that reporter activity was unchanged, confirming that serum is not a source of norrin (Figure 8D). Norrin-induced signaling in TOPFlash reporter assays is substantially higher than basal receptor signaling (10). The relatively low activity of basal signaling compared with ligand-induced signaling likely explains why the additional increase in BRB leakage and CE formation in *Tspan12*-KO^DBM^; *C1qa*-KO compound mutant mice is moderate, and why *C1qa*-single-KO mice (which maintain strong norrin-induced FZD4 signaling) have no obvious BRB phenotypes. Together, our data indicate that C1q-mediated activation of β-catenin–dependent signaling provides basal activity that is important in the context of a dysfunctional BRB. Disease contexts in which this mechanism is likely relevant include familial exudative vitreoretinopathy (FEVR), which is a retinal vascular disease caused by impaired norrin/FZD4 signaling, for example by mutations in the human *TSPAN12* gene (42).

### F4L5.13 achieves complete resolution of CE in treatment-naive Tspan12-ECKO mice.

**Figure 9:**
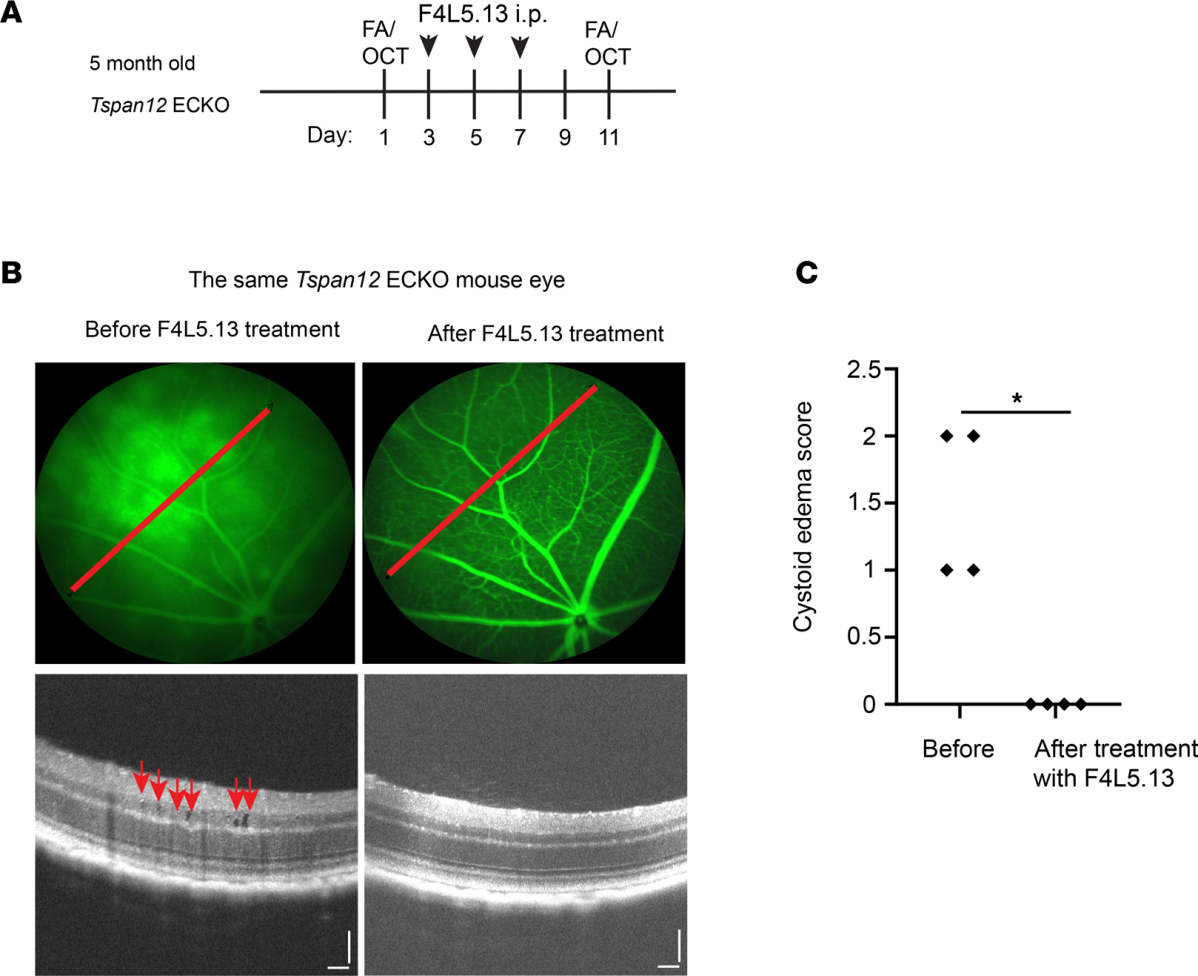
F4L5.13 achieves complete resolution of CE in treatment-naive *Tspan12*-ECKO mice. (**A**) Schematic representation of the experimental design. Animals received 3 doses of F4L5.13, 10 mg/kg, i.p. (**B**) Representative FA fundus images and OCT scan images show *Tspan12*-ECKO mice with retinal vascular leakage and moderate CE lesions. The red lines show the OCT line scan relative to the FA image. Right panels show the same retina after treatment. Scale bars: 100 μm. (**C**) A Mann-Whitney nonparametric test was used to test for differences of CE scores on an ordinal scale. *n* = 4 retinas. *P < 0.05.

We previously reported that F4L5.13 restores BRB function in *Tspan12*-ECKO mice (18); therefore, we wondered whether the norrin mimetic can also alleviate CE. We used the *Tspan12*-ECKO model for this experiment, as these mice are treatment naive. Three doses of F4L5.13 were administered to 5-month-old *Tspan12-*ECKO mice i.p. every 2 days (Figure 9A). Image-guided OCT showed moderate CE before treatment. The same mouse eye was reimaged 11 days later. Strikingly, F4L5.13 treatment rescued BRB leakage and CE was completely resolved in all retinas (Figure 9, B and C). This finding demonstrates the efficacy of FZD4/LRP5 agonists in a mouse model of CE and reinforces the concept that the norrin/FZD4 signaling pathway is a highly suitable target for pharmacological intervention in ME.

## Discussion

A major conclusion from the present study is that loss of C1QA, which is required for the initiation of the classical complement cascade, reduces basal β-catenin–dependent signaling activity in ECs and exacerbates BRB dysfunction in a context of impaired norrin/FZD4 signaling. This conclusion is based on phenotypic comparison of *Tspan12*-KO^DBM^; *C1qa*-KO compound mutant mice, cell-based assays for basal FZD4/LRP5 signaling, as well as prior studies that established a relationship between C1q and basal frizzled signaling in other biological contexts (33, 34). The increased BRB dysfunction is associated with increased formation of CE and increased neuroinflammation. The role of C1q in BRB function is likely relevant in the context of FEVR and Norrie disease, related inherited diseases caused by impaired norrin/FZD4 signaling. The genes mutated in these diseases include *NDP* (norrin) in Norrie disease and *FZD4*, *LRP5*, and *TSPAN12* in FEVR (42). Our data indicate that C1q maintains a degree of β-catenin signaling in a FEVR mouse model, and that loss of C1q exacerbates BRB defects and neuroinflammation in this model. A role for C1q as a factor in BRB or blood-brain barrier maintenance may extend to other retinal and neurological diseases beyond FEVR, including diabetic retinopathy and stroke.

Inherited deficiency of C1QA is a major risk factor for SLE development (43), which may lead to lupus retinopathy (41). C1QA deficiency is thought to promote autoimmunity by impairing the clearance of apoptotic cells and nuclear antigens, which may cause the generation of autoantibodies. *C1qa*-KO mice develop autoantibodies on specific genetic backgrounds, causing reduced long-term survival (35, 44). We performed our aging study on a C57BL/6J background that promotes survival but limits the development of autoimmunity. Potentially, the strong immunoglobulin extravasation in *Tspan12*-KO^DBM^; *C1qa*-KO compound mutant mice would promote autoimmune responses and lupus retinopathy with cotton-wool spots, and hemorrhages. The finding that these phenotypes were not present in *Tspan12*-KO^DBM^; *C1qa*-KO mice suggests that autoimmunity does not develop. Reasons include the genetic background, which may affect immune responses, or that cellular debris is still sufficiently cleared even in the absence of C1QA. *C1qa*-deficient microglia may use alternative pathways to clear cellular debris. Potential alternative find-me signals, eat-me signals, or opsonins include IgG, complement components C3b and C4b, pentraxins, or ApoE. Microglia may use Fcγ receptors, complement receptors C3AR1 and C5AR1, or other receptors to detect these signals in a C1q-independent manner (45). Indeed, Fcγ receptors, complement receptors, and complement components including C4b are strongly upregulated in *Tspan12* ECKO with BRB dysfunction (7). Furthermore, the expansion of the microglia/monocyte-derived macrophage population may compensate for any reduction in the cellular rate of phagocytosis in *Tspan12-* KO^DBM^; *C1qa*-KO microglia/macrophages. In addition to immune cells, astrocytes may phagocytose targets that are opsonized by C4b (40).

*Tspan12*-KO^DBM^ and *Tspan12*-KO^DBM^; *C1qa*-KO compound mutant mice display a strong reduction in the ERG b-wave. The reduction in the ERG b-wave in treatment-naive conventional *Tspan12*-KO mice, which are exposed to hypoxia in the inner nuclear layer due to angiogenesis defects (18), is only moderately more severe than the reduction in the ERG b-wave in *Tspan12*-KO^DBM^ mice with normal angiogenesis and normoxia that we used in this study. While hypoxia likely contributes to the reduction in the ERG b-wave in conventional *Tspan12*-KO mice, our findings highlight that BRB dysfunction also disturbs retinal homeostasis in a hypoxia-independent manner, for example by altering K^+^ ion homeostasis (46).

A second major conclusion from this study is that F4L5.13 agonist achieves complete resolution of CE in treatment-naive *Tspan12-*ECKO mice. FZD4/LRP5 agonists also reduce neovascular tufts in mice with oxygen-induced retinopathy (16, 47); therefore, this class of agonists appears to target multiple processes relevant to diabetic retinopathy, including promoting BRB function and reducing pathological neovascularization. Our observation that F4L5.13 resolves CE further validates the norrin/FZD4 signaling pathway as a compelling target for drug development in ME, for example in diabetic retinopathy, age-related macular degeneration, or retinal occlusive disease.

## Methods

### Sex as a biological variable.

Animals of both sexes were used for all studies. The study was not designed or powered to detect sex differences.

### Animals.

*Tspan12*-floxed (*Tspan12*^tm1.1Hjug^) and -null (*Tspan12*^tm1.2Hjug^) alleles were reported previously (7). Tg(Cdh5-cre/ERT2)1Rha (48) was used as a EC-specific Cre driver and was provided by Ralf Adams Max Planck Institute for Molecular Biomedicine, Munster, Germany) under an MTA with CancerTools (https://cancertools.org/). *C1qa*-KO mice were obtained from Laura Nagy (Cleveland Clinic, Cleveland, Ohio, USA)with permission from the owner of the material, Marina Botto (Imperial College London). To ensure genetic consistency and survival of *C1qa*-KO mice to the T_0_ + 12-month endpoint, all strains were backcrossed at least 7 generations with C57BL/6J mice. All mice were housed in a specific pathogen–free animal facility.

### F4L5.13 antibody administration.

F4L5.13 (16) or vehicle (10 mM histidine, 0.9% sucrose, 150 mM NaCl, pH 6.0) was administered i.p. to *Tspan12-*KO^DBM^ pups at 10 mg/kg on P6, P9, and P12, and 4 mg/kg on P15, P18, P21, P24, and P27. Adult mice received multiple doses of 10 mg/kg, i.p., as indicated in the respective figures.

### FA.

Mice were anesthetized using an isoflurane gas delivery system. Immediately after anesthesia, pupils were dilated with a 1:1 mix of 1% tropicamide and 2.5% phenylephrine, administered as an eyedrop. Systane gel was applied to the cornea. Fluorescein (final 0.25%, diluted in 0.9% sterile saline from 10% fluorescein-sodium, Akorn) was administered subcutaneously at 10 μL/g bodyweight. Fluorescent fundus images were acquired 5 minutes after the fluorescein administration. A Micron III small animal fundus imaging system was used to acquire images. When FA was performed in the context of OCT, a Micron IV image-guided OCT system was used (Phoenix Research Laboratories). Mice were placed on a heating pad during recovery from anesthesia.

### Image-guided OCT.

Mice were prepared for the experiment as described under *FA*. Ten OCT line scans were acquired, typically in the superior temporal quadrant as indicated by the red lines in FA images obtained during image-guided OCT. Line scans were acquired with a scan length of 1.45 mm and a spacing of 15 pixels. Images were captured using RevealOCT 2.1.6 software (Phoenix). Image processing (sharpening and contrast enhancement) was performed in ImageJ (NIH). CE was scored against the grading scale described in Supplemental Figure 1 in a blinded fashion.

### Electroretinography.

ERGs were acquired under red light after overnight dark adaption. Mice were anesthetized using an isoflurane gas delivery system, pupils were dilated with a 1:1 mix of 1% tropicamide and 2.5% phenylephrine, administered as eyedrop, and hypromellose lubricant eye gel (Systane) was applied to both corneas. ERG recordings were obtained on a heated platform of a Celeris Diagnosys ERG system. The impedance ranged from 5 to 15 KΩ. The eyes received stimulation at 1 cd•s/m^2^.

### Sulfo-NHS-LC-biotin labeling.

Sulfo-NHS-LC-biotin (250 μL of 20 mg/mL) (Life Technologies, 21335) was injected i.p. After 60 minutes, mice were anesthetized using an isoflurane drop jar, euthanized by cervical dislocation, and immediately transcardially perfused with 2 U/mL heparin in PBS.

### Whole-mount retinal staining and quantification.

Mouse eyes were dissected immediately after transcardial perfusion and mildly fixed in 4% PFA at room temperature (RT) for 15 minutes. After 3 washes with PBS, the retinas were dissected and blocked for 1 hour at RT with blocking buffer (5% goat serum, 0.5% Triton X-100 in PBS). Retinas were stained with streptavidin–Alexa Fluor 488 (Invitrogen, S11223) or anti-IBA1 (Cell Signaling Technology, 17198) or anti-CD11b (BD, 550282) in blocking buffer overnight at 4°C. Retinas were washed 6 times (30 minutes/wash) at RT in PBS with 0.5% Triton X-100. IBA1 staining was completed with secondary goat anti-rabbit–Alexa Fluor 488 (Life Technologies, A11008) in blocking buffer overnight at 4°C. Images were acquired using a Keyence BZ-X800, and ImageJ was used for the quantification. For streptavidin quantification, ×4-magnified images were stitched. A threshold value was defined for the streptavidin-positive blood vessels of WT retinas, and then this threshold value was used for all other groups to obtain the streptavidin-positive area above the threshold. In addition, streptavidin raw fluorescence intensity was quantified from the ImageJ mean gray value per retinal area. For the quantification of IBA1-positive cells, image stacks were acquired at ×20 magnification, and a maximum intensity projection was generated. IBA1-positive cells were manually counted from the projections. CD11b raw fluorescence intensity was quantified from the ImageJ mean gray value per ×20 field of view.

### Retinal sections staining and quantification.

Unfixed retinal sections were cut as described previously (49). In brief, eyes were enucleated and rapidly embedded unfixed in optimal cutting temperature compound (Tissue-Tek) using a cryomold, which was placed into a container of 2-methylbutane cooled in liquid nitrogen. Tissue blocks were cryosectioned at 12 μm thickness, mounted onto Superfrost Plus slides, air-dried briefly, and postfixed in 4% paraformaldehyde (PFA) for 15 minutes at room temperature. Sections were then permeabilized and blocked in a solution of 5% goat serum with 0.5% Triton X-100 for 30 minutes at ambient temperature. Primary labeling was performed using anti-mouse IgG (Thermo Fisher Scientific, A11001) for 1 hour at RT. The following day, sections were rinsed 5 times for 5 minutes each in PBS containing 0.1% Triton X-100. Fluorophore-conjugated secondary antibodies were applied for 1 hour at RT. Finally, slides were coverslipped with FluoroMount-G mounting medium (SouthernBiotech, 0100-01), and images were captured using a Keyence BZ-X810 digital imaging system. IgG raw fluorescence intensity was quantified from the ImageJ mean gray value per retinal region of interest, which encompassed the cropped retina without adjacent extraretinal tissue. TUNEL was performed on retinal sections using a kit from Promega (G3250). As positive control, a dissected unfixed eyeball was incubated in high-glucose DMEM (Corning, 10-013-CV) with 5 mM *tert*-butyl hydroperoxide at 37°C for 1 hour before sectioning as described above.

### Quantitative PCR.

RNA was extracted from mouse retinas using RNAzol-RT (ABP Bioscience, FP314) according to the manufacturer’s instructions. Equal quantities of RNA were reverse transcribed into cDNA using the Maxima First Strand cDNA Synthesis Kit (Thermo Fisher Scientific, K-1642). Quantitative PCR (qPCR) was conducted with SYBR green detection and data analysis was performed using the ΔΔCt method. Primers for qPCR were designed to span exon-exon junctions. The following primers were used — mGapdh Forward: 5′-GGGTGAGGCCGGTGCTGAGT-3′ and Reverse: 5′-TCGGCAGAAGGGGCGGAGAT-3′; mTrem2 Forward: 5′-CGAGGGTGCCCAAGTGGAAC-3′ and Reverse: 5′-GGTGGTAGGCTAGAGGTGACCC-3′; mC4b Forward: 5′-GGCACACCTTGCCCGAAACA-3′ and Reverse: 5′-AACCAAGCCCCAAAGGAGCC-3′; mAqp4 Forward: 5′-GCTCGATCTTTTGGACCCGCA-3′ and Reverse: 5′-GCACAGCGCCCATGATTGGT-3′.

### Luciferase reporter assay and collection of serum.

293T cell suspensions (0.4 mL of 330,000 cells/mL) in high-glucose DMEM with 5% mouse serum of the indicated genotype were seeded into each well of a 48-well plate. After 6 hours, cells were transfected with 160 ng DNA (4 ng FZD4, 8 ng LRP5, 8 ng GFP or TSPAN12, and 140 ng reporter mix that was composed of TOPFlash plasmid, CMV-Renilla, and Lef1 as described previously; ref. 10) using TransIT-LT1 (Mirus). Eighteen hours later, DualGlo luciferase assays were performed, and firefly and Renilla luciferase signals were measured using a Synergy LX Multimode Reader (Agilent BioTek). The ratio of firefly/Renilla luciferase signals was calculated, and data were normalized to the reading obtained with WT mouse serum. To collect mouse serum, blood was withdrawn slowly via the left ventricle of deeply anesthetized mice using a 26-gauge needle. Mice were euthanized after blood collection. The blood samples sat undisturbed at RT for 30 minutes to allow clotting. Samples were spun at 2,000*g* for 10 minutes at RT. The clear supernatant (serum) was frozen.

### Western blot.

For Western blot analysis of serum, 2 μL of serum was loaded per lane. An XCell SureLock gel electrophoresis and NuPAGE 4× LDS sample buffer, MES running buffer, and transfer buffer were used (Novex, NP0008, NP0002, and NP0006). Nitrocellulose membranes were probed with Ponceau S to document total protein load and with anti-C1QA (Abcam, EPR28764-66; 1:1000) and Rabbit Trueblot Ultra (Rockland, 18-8816-31; 1:1000).

### Statistics.

A Shapiro-Wilk test for normality and Levene’s test for homogeneity of variance was performed. Parametric or nonparametric 2-group and multigroup comparisons were performed as described in each figure legend. Parametric tests were homoscedastic or heteroscedastic *t* tests or 1-way ANOVA with Tukey’s post hoc test; nonparametric tests were Mann-Whitney or Kruskal-Wallis tests. CE scores are on an ordinal scale; therefore, these data were tested using a nonparametric test (50). Because CE and leakiness were markedly different between the 2 eyes of 1 mouse, we evaluated each retina separately without averaging. A *P* value of less than 0.05 was considered significant.

### Study approval.

All animal protocols were approved by the Animal Care and Use Committee of the University of Minnesota, Twin Cities.

### Data availability.

All data supporting the findings of this study are available within the article and its supplemental data and Supporting Data Values file.

## Author contributions

LZ and HJJ wrote the manuscript. HJJ designed the study. LZ, JL, MA, HNJ, MH, EO, KLD, ET, and HJJ conducted experiments. SWM, HR, and ZC trained personnel or provided the required instrumentation. SS and SA provided the F4L5.13 antibody.

## Funding support

This work is the result of NIH funding, in whole or in part, and is subject to the NIH Public Access Policy. Through acceptance of this federal funding, the NIH has been given a right to make the work publicly available in PubMed Central.

- NIH grants R01EY024261 and R01EY033316 (to HJJ).
- NIH grant R21DA056728 (to ZC).
- Canadian Institute of Health Research grant PJT-175160 (to SA).

## Supplementary Material

Supplemental data

Unedited blot and gel images

Supporting data values

## Version 1 10/14/2025

In-Press Preview

## Version 2 11/24/2025

Electronic publication
