# Supplementary material for: C1q limits cystoid edema by maintaining basal β-catenin–dependent signaling and blood-retina barrier function
Source: JCI Insight. 2025 Oct 14;10(22):e190227. doi: 10.1172/jci.insight.190227 (PMC12643494; doi:10.1172/jci.insight.190227)
Supplement: Supplemental data [file jciinsight-10-190227-s047.pdf]

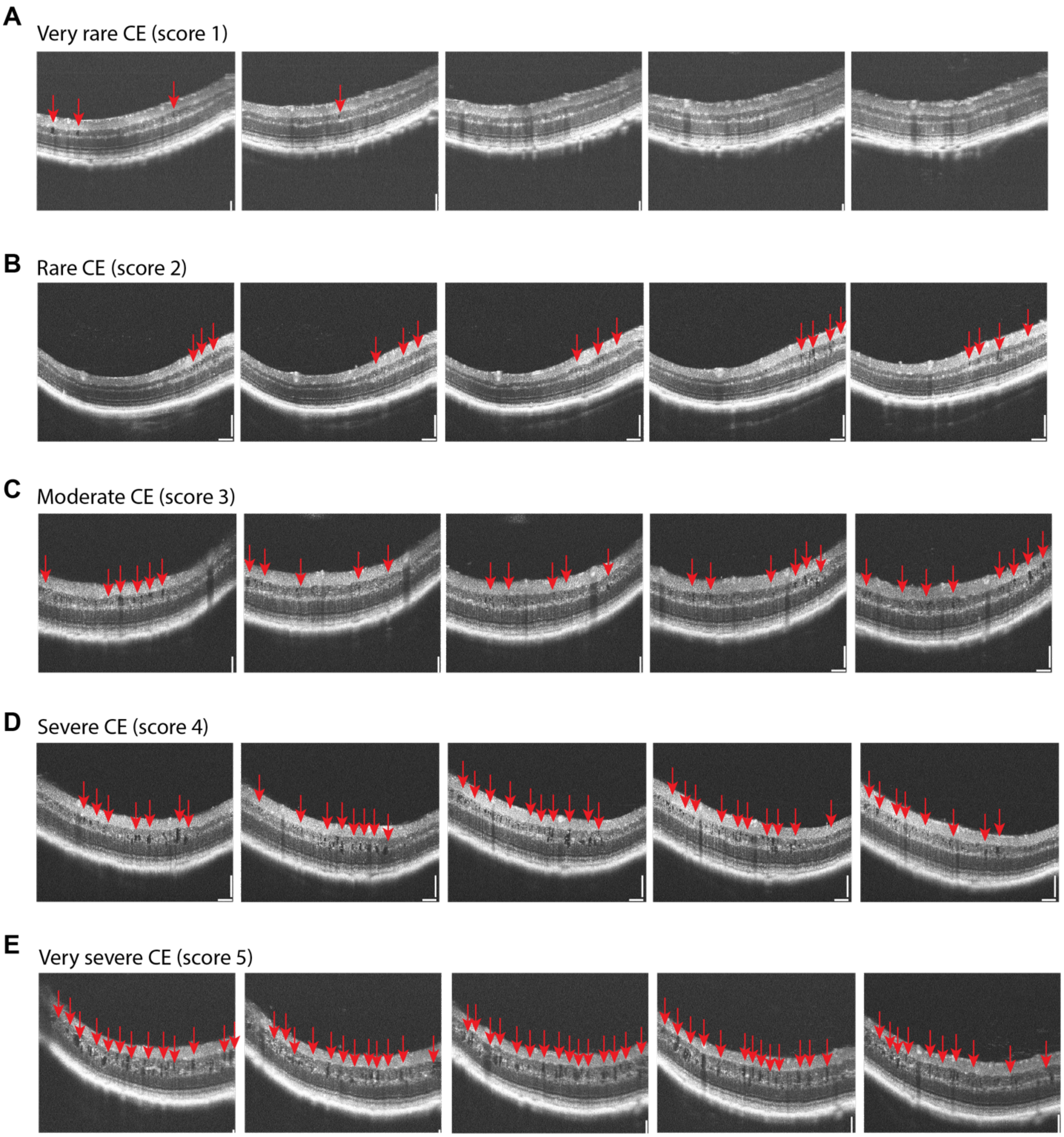


Supplemental Figure S1. Representative OCT line scans representing CE of increasing severity, scored with 1 (very rare CE) to 5 (very severe CE). Five adjacent OCT line scans are shown per retina. Scale bars (100 µm) are not fully shown in all images due to cropping, the scale bars in panel B apply to all images.

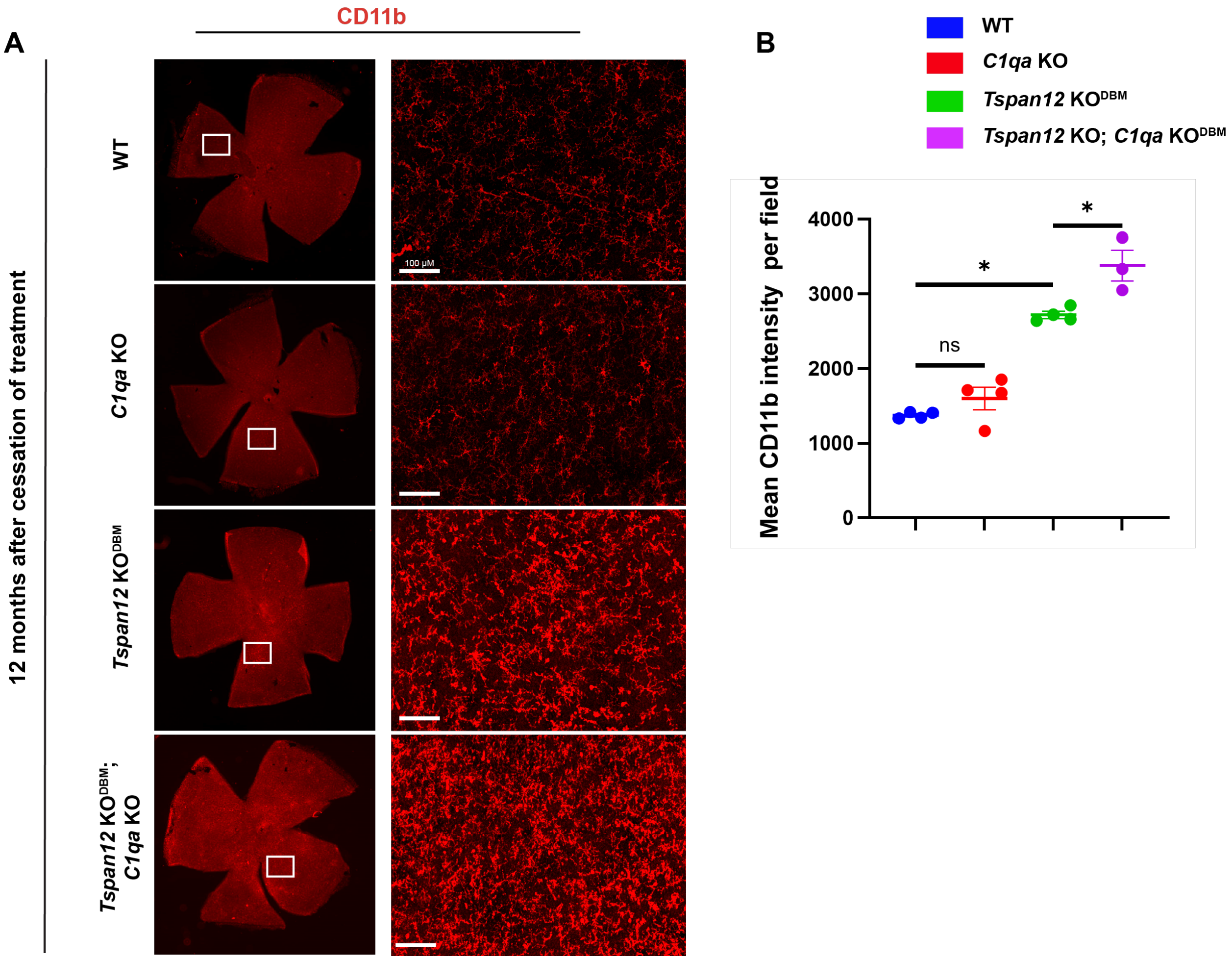


Supplemental Figure S2. (**A**) Retinal wholemounts were stained for the myeloid cell marker CD11b. Scale bar: 100 µm. (**B**) Average intensity projections from 4 fields of view per retina were analyzed for CD11b raw fluorescence intensity and averaged. N=3-4 retinas from 3-4 mice, one-way ANOVA with Tukey post-hoc, average +/- SEM shown. * indicates P<0.05.

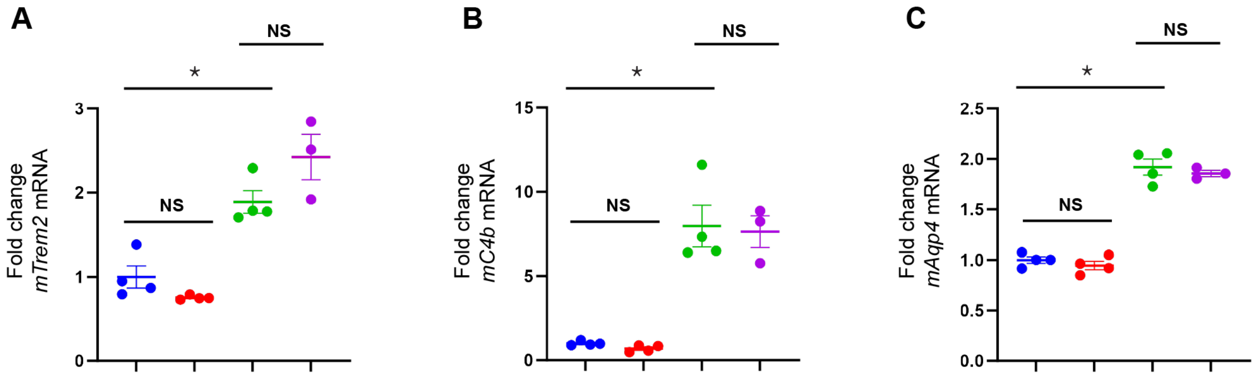


Supplemental Figure S3. (**A-C**) RT-qPCR for the indicated genes, N=3-4 retinas per group, one-way ANOVA with Tukey post hoc, average +/- SEM shown.

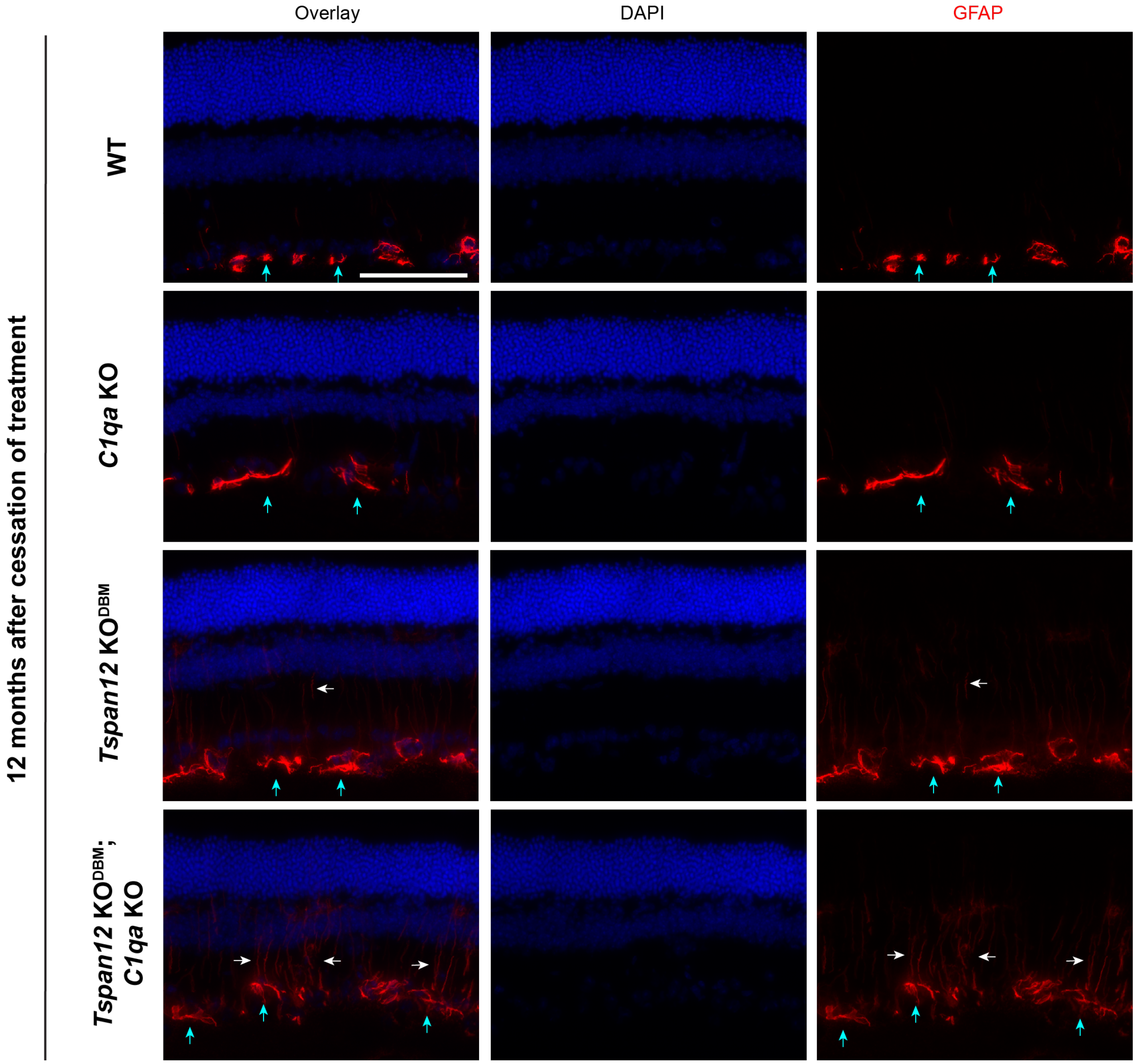


Supplemental Figure S4. Retinal sections from mice at the study endpoint were probed with anti-GFAP. Three retinas from three mice per genotype were stained and imaged with similar results. GFAP was strongly expressed in retinal astrocytes (blue arrows) in all genotypes. In *Tspan12 KO$^{DBM}$* and *Tspan12 KO$^{DBM}$*; *C1qa* KO compound mutant retinas, GFAP was additionally detected in thin Müller glia processes (white arrows), indicating reactive gliosis.

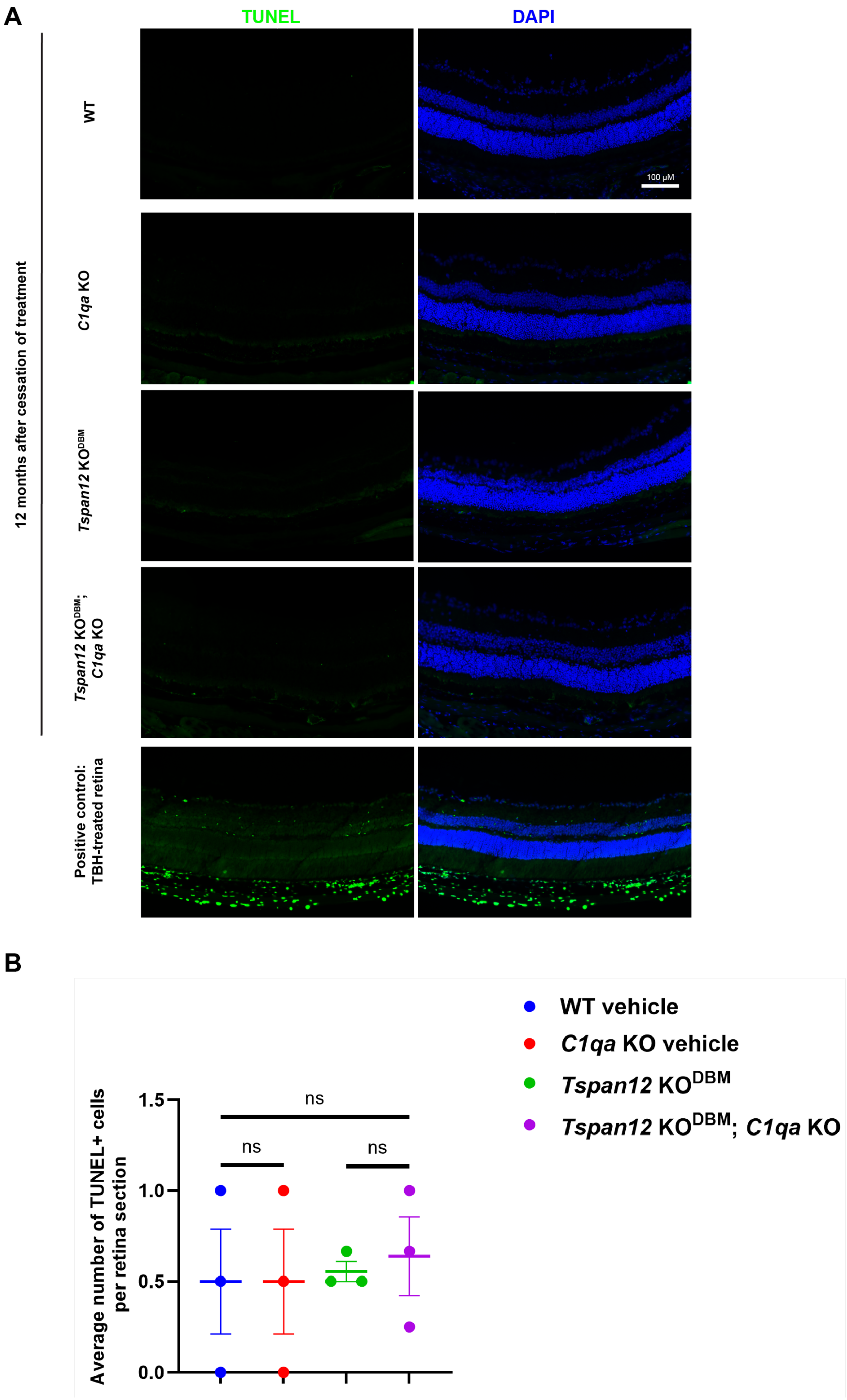


Supplemental Figure S5. No substantial ongoing apoptosis 12 months after cessation of treatment. (**A**) TUNEL performed on retinal sections, scale 100 µm. (**B**) Quantification of TUNEL$^{+}$ cells per 20x field of view. Two fields of view per retina were averaged, N=3 retinas from 3 mice, one-way ANOVA with Tukey post-hoc, average +/- SEM shown.

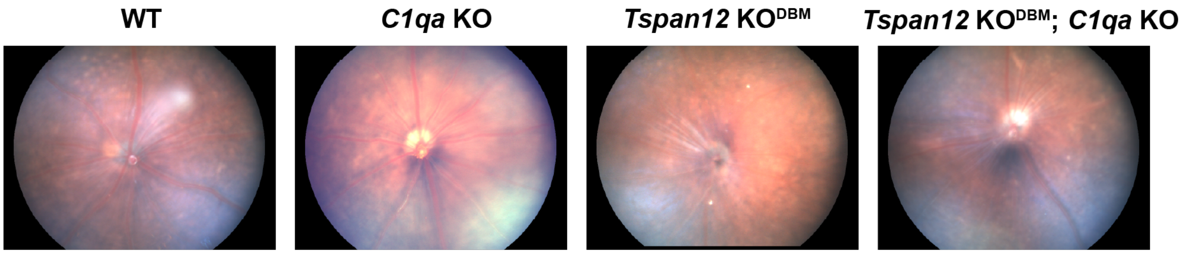


Supplemental Figure S6. No hemorrhages or cottonwool spots in *Tspan12* KO$^{DBM}$; *C1qa* KO mice. Representative fundus images of N=3 mice per group. The images are focused on the surface of the retina to allow detection of potential cotton-wool spots. SLE retinopathy was not detected in any of the four genotypes.
