# Supplementary material for: C1q limits cystoid edema by maintaining basal β-catenin–dependent signaling and blood-retina barrier function
Source: JCI Insight. 2025 Oct 14;10(22):e190227. doi: 10.1172/jci.insight.190227 (PMC12643494; doi:10.1172/jci.insight.190227)

Fig. 8A

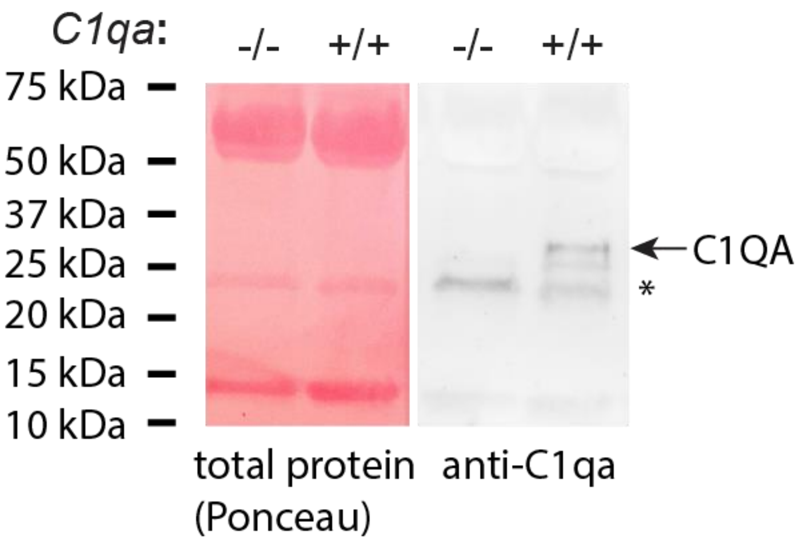

Original data, n=3 per group. Arrows show representative lanes that were rotated and cropped out

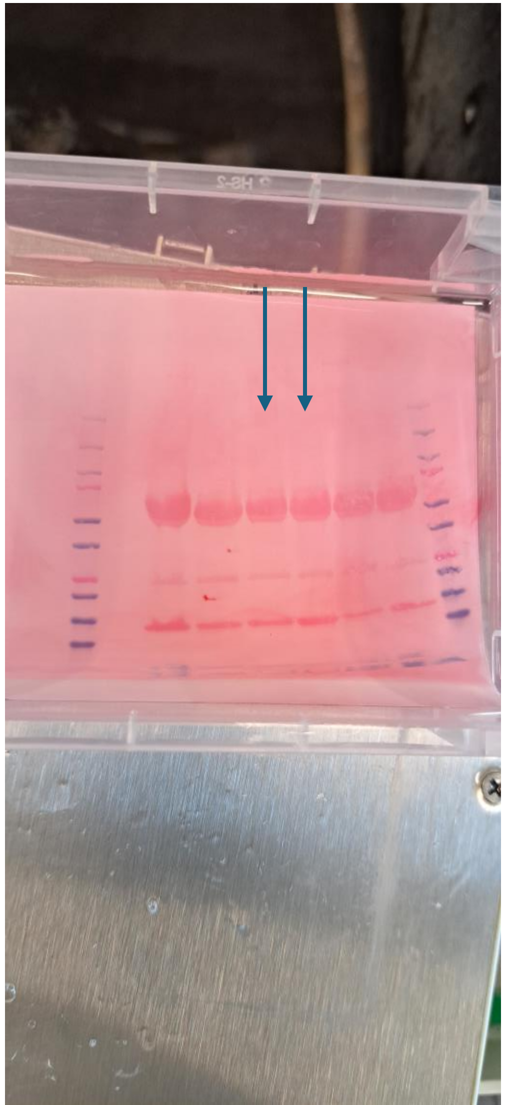

Original data, n=3 per group. Arrows show representative lanes that were rotated and cropped out

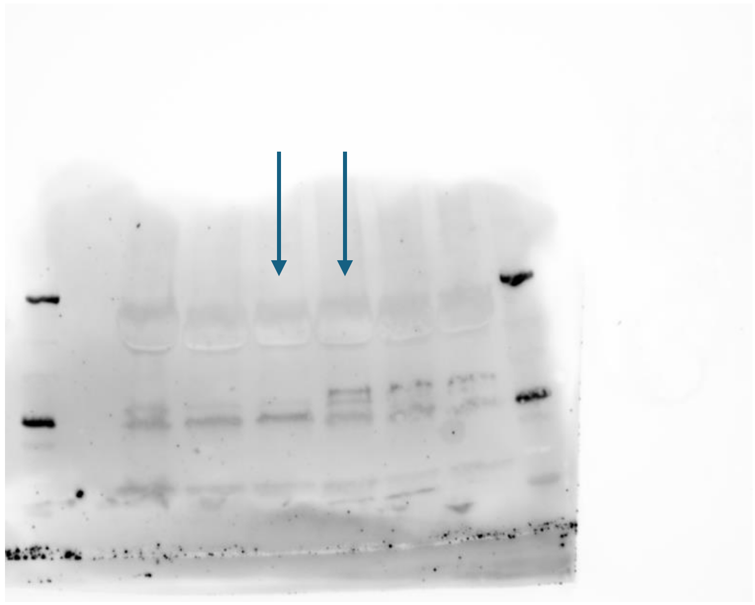

Supplement: Unedited blot and gel images [file jciinsight-10-190227-s048.pdf]
